# Environmental and seasonal correlates of capercaillie movement traits in a Swedish wind farm

**DOI:** 10.1002/ece3.7922

**Published:** 2021-08-05

**Authors:** Jim‐Lino Kämmerle, Julia Taubmann, Henrik Andrén, Wolfgang Fiedler, Joy Coppes

**Affiliations:** ^1^ FVA Wildlife Institute Forest Research Institute of Baden‐Wuerttemberg FVA Freiburg Germany; ^2^ Chair of Wildlife Ecology and Management University of Freiburg Freiburg Germany; ^3^ Grimsö Wildlife Research Station Department of Ecology Swedish University of Agricultural Sciences Riddarhyttan Sweden; ^4^ Department of Migration and Immuno‐Ecology Max Planck Institute of Animal Behavior Radolfzell Germany

**Keywords:** GPS telemetry, grouse, HMM, movement speed, step analysis, step length, Tetrao urogallus

## Abstract

Animals continuously interact with their environment through behavioral decisions, rendering the appropriate choice of movement speed and directionality an important phenotypic trait. Anthropogenic activities may alter animal behavior, including movement. A detailed understanding of movement decisions is therefore of great relevance for science and conservation alike. The study of movement decisions in relation to environmental and seasonal cues requires continuous observation of movement behavior, recently made possible by high‐resolution telemetry. We studied movement traits of 13 capercaillie (*Tetrao urogallus*), a mainly ground‐moving forest bird species of conservation interest, over two summer seasons in a Swedish windfarm using high‐resolution GPS tracking data (5‐min sampling interval). We filtered and removed unreliable movement steps using accelerometer data and step characteristics. We explored variation in movement speed and directionality in relation to environmental and seasonal covariates using generalized additive mixed models (GAMMs). We found evidence for clear daily and seasonal variation in speed and directionality of movement that reflected behavioral adjustments to biological and environmental seasonality. Capercaillie moved slower when more turbines were visible and faster close to turbine access roads. Movement speed and directionality were highest on open bogs, lowest on recent clear‐cuts (<5 y.o.), and intermediate in all types of forest. Our results provide novel insights into the seasonal and environmental correlates of capercaillie movement patterns and supplement previous behavioral observations on lekking behavior and wind turbine avoidance with a more mechanistic understanding.

## Graphical abstract

We analyzed movement traits (speed and directionality) of capercaillie, a mainly ground‐dwelling forest bird, in a Swedish wind farm. We found evidence for clear daily and seasonal variation in movement traits that reflected behavioral adjustments to biological and environmental seasonality. Capercaillie movement behavior was also related to wind turbine effects, mainly turbine visibility, and habitat characteristics.

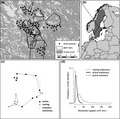

## 1 INTRODUCTION

Animal movement is a fundamental property of biological systems, shaping their structure and dynamics from individual behavioral decisions to the community or ecosystem level (Joo et al., 2020; Nathan et al., 2008). The decision whether to move and at what speed is nontrivial and governs an animal's behavioral interaction with its environment (Wilson et al., 2015). Movement has aptly been called the “glue” connecting occurrence and behavior (Van Moorter et al., 2016). The appropriate choice of movement speed and directionality may therefore be linked with individual animal performance, rendering it an important phenotypic trait (Ciuti et al., 2012; Wilson et al., 2015). Its detailed observation may thus be of great relevance for science and conservation alike. Examples include patterns in migration and dispersal (Cagnacci et al., 2011; Killeen et al., 2014; Walton et al., 2018), habitat selection in response to environmental cues (Fortin et al., 2005; Roever et al., 2010; Taubmann et al., 2021), identifying movement corridors (Chetkiewicz et al., 2006; Squires et al., 2013; Thirgood et al., 2004) or witnessing inter‐specific interactions (Dröge et al., 2017; Eriksen et al., 2009).

The study of animal movement has been revolutionized by animal‐borne tags (Cagnacci et al., 2010; Morales et al., 2004; Tomkiewicz et al., 2010), particularly with the advent of GPS tags (Tomkiewicz et al., 2010). However, despite a new frontier in the tracking of very small animals (e.g., Fisher et al., 2020; Kissling et al., 2014), fine‐scale analyses of movement behavior remain relatively infrequent for terrestrial mammals and birds (e.g., sampling rates <1 hr intervals) and have mostly been performed for larger‐bodied or highly mobile species (e.g., Reusch et al., 2020; Thurfjell et al., 2014; but see, e.g., Gillies et al., 2011; McDuie et al., 2019). Nonetheless, animals continuously interact with their environment through behavioral decisions (e.g., small‐scale resource selection, feeding, predator avoidance), which may be undetectable at the typical resolution of movement analyses (e.g. hourly‐daily; Thurfjell et al., 2014). Advances in spatial and temporal resolution can thus greatly benefit the study of animal behavior by closing the gap between sampling regime and the rate at which behavioral decisions are taken. Modern tracking technology thus holds great potential (Cagnacci et al., 2010; Hebblewhite & Haydon, 2010).

The study of movement traits and their relationship with the environment ideally requires continuous observation of behavioral decisions, which is a daunting task for secretive species and in remote or dense habitats. Forest grouse (*Galliformes, Tetraoninae*) are one such example. Grouse are large ground‐nesting birds with 20 species occurring at temperate and boreal latitudes across the northern hemisphere (IUCN, 2020). They are popular game birds and, in many places, of high conservation interest owing to declining trends in reproductive success and abundance (Jahren et al., 2016; Storch, 2007). Behavioral reactions of grouse to wind turbines have been reported by several studies (Coppes, Braunisch, et al., 2020; Coppes, Kämmerle, et al., 2020; Taubmann et al., 2021). Notwithstanding a historically large scientific interest in the study of grouse population parameters, behavioral studies remain comparatively rare, owing to the difficulty of observing their behavior in the field. As a forest‐living species, their movement in particular is very difficult to observe. Perhaps unsurprisingly, many existing studies thus infer conservation‐relevant spatial behaviors from indirect data or behavior at the lek, such as avoidance of anthropogenic disturbance caused by wind turbines or recreational activities (e.g., Coppes, Braunisch, et al., 2020; Coppes, Kämmerle, et al., 2020; Coppes et al., 2018; Summers et al., 2007), while the underlying behavioral decisions remain largely unknown. In addition, while habitat selection studies using VHF‐tags have provided information on habitat selection using single instants in time (e.g., Brøseth & Pedersen, 2010; Storch, 1993, 1995; Thiel et al., 2008), modern tracking technology enables a more mechanistic understanding of the movement decisions underlying habitat selection patterns.

Here, we analyze movement traits of capercaillie (*Tetrao urogallus*), a primarily ground‐dwelling and slow‐moving grouse species inhabiting coniferous forests, in two adjoining Swedish wind farms using GPS data collected at a high temporal resolution (defined as a 5‐min sampling interval) by means of a two‐step approach. We first filtered potentially noisy movement data using classification of activity and movement state based on accelerometer data and step characteristics in order to then study capercaillie movement traits. We focused on (1) environmental and (2) seasonal variation in fine‐scale movement behavior (i.e., movement speed and directionality) and (3) explored behavioral reactions to wind turbines.

## 2 MATERIAL AND METHODS

### 2.1 Study area and species

The capercaillie is a forest grouse with a wide distribution across the boreal and montane forests of Eurasia. Owing to their wide distributional range, they are not threatened at the global scale (Birdlife‐International, 2016). However, abundance and reproductive success have been locally declining (Jahren et al., 2016) and many populations are red‐listed, particularly in Central Europe. Capercaillie prefer mature, well‐structured, and conifer‐dominated forests with canopy gaps, stand edges, and ground vegetation dominated by bilberry (*Vaccinium myrtillus*) (Braunisch et al., 2014; Graf et al., 2009; Klaus et al., 1989). In addition to mature forest stands, capercaillie select other stands with suitable structures such as open stands, stand edges, canopy gaps, or single large trees (Hofstetter et al., 2015; Klaus et al., 1989; Storch, 1991, 1993). They have been proposed as an umbrella species for structurally diverse, species‐rich conifer‐dominated forests (Pakkala et al., 2003; Suter et al., 2002). Capercaillie, like most grouse, often walk on the forest floor and prefer to glide (e.g., down a mountain slope), rather than fly actively (Klaus et al., 1989; Wegge et al., 2013).

**FIGURE 1:**
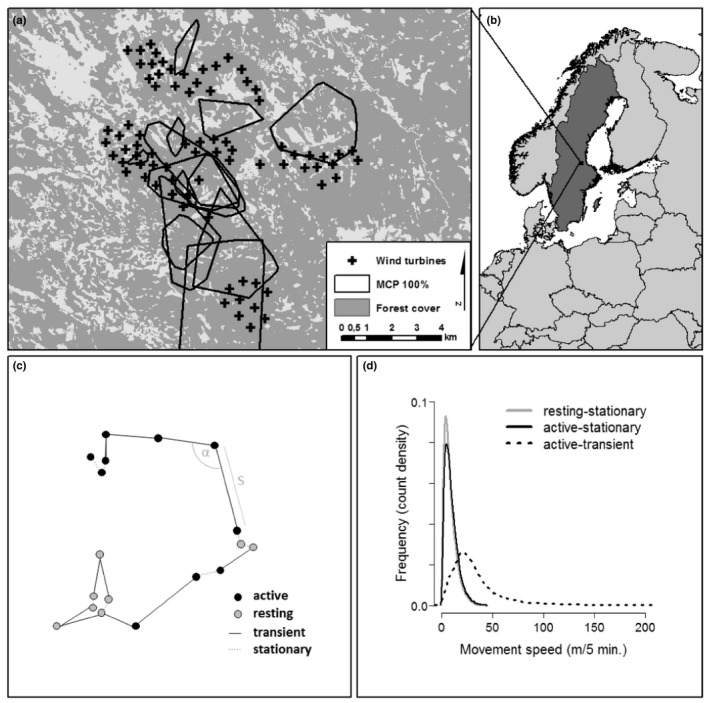
Home ranges of GPS‐tagged capercaillie that delivered high‐resolution movement data in the study area (a), that was located in Sweden (b). The study was conducted in two adjoining wind farms, Mombyåsen (10 southernmost turbines) and Jädraås (north). GPS fixes within regular movement bursts (5‐min sampling interval) were classified as belonging to an *active* or *resting* behavioral state using 3D‐accelerometer data and movement steps were classified to represent *stationary* or *transient* states based on step length *S* and turning angle α (c). Accuracy filtering resulted in the exclusion of steps classified as “*resting*–*transient*,” which were considered to have a high probability of large GPS positional error while the animal was in fact stationary. This resulted in three types of movement steps considered in this analysis (d)

The study was conducted in two adjoining wind farms in central Sweden (Figure 1a,b) in Dalarna and Gävleborg County. The size of the study area was approx. 10,000 ha and included the Jädraås wind farm with 68 Vestas V112 turbines and the Mombyåsen wind farm with 10 Vestas V126 turbines. The study area is predominantly forested, with Scots pine (*Pinus sylvestris*), Norway spruce (*Picea abies*), and, partly, silver birch (*Betula pendula*) forest stands subject to clear‐cut forestry that are fragmented by open bogs, lakes, and settlements (Figure 1a). The terrain is characterized by gentle hills (elevation range of the study area: 65–465 m a.s.l.). Capercaillie occur throughout the study area.

### 2.2 Animal capture and transmitter fitting

Adult capercaillie were captured at active lekking sites in the wind farm in 2017 and 2018 (in mid to late April) using walk‐in nets and, later in the season (May), at sand baths. Birds were tagged and released after a maximum handling time of 10 min. To maximize sampling duration and minimize the sampling interval, we used both solar and battery GPS‐3D‐acceleration transmitters (Bird 1AA2, Bird 1A‐light, Bird Solar and Bird 2AA2, E‐obs digital telemetry, Munich, Germany). Solar tags weighed 38 g and were used for both sexes. Battery tag weight was 38 g (female) to 48 g (male), which is up to 2% of the adult birds´ total weight. A total of 18 adult birds (12 males, 6 females) were fitted with GPS tags, of which 13 were fitted with solar tags, which enabled a five‐minute GPS sampling schedule given sufficient battery power. All tags were also programmed to record high‐resolution acceleration data every 3 min for 10 s with a frequency of 20 Hz for all three axes. The data were downloaded from the tags at regular intervals (at least monthly) using a handheld device, at a distance of several hundred meters. For subsequent analysis, we only used high‐resolution GPS data on animal movement behavior and therefore selected only those GPS position fixes (henceforth: “*fixes”*) collected on a five‐minute sampling schedule. We thus obtained a total of 184,004 fixes from 13 animals collected between 20 April 2017 and 27 August 2018. We removed all GPS fixes obtained within three days after capture to exclude behavior potentially affected by the capture events.

### 2.3 Classification of movement behavior and filtering

We classified all GPS fixes that were successfully taken on a regular sampling schedule (i.e., every five minutes) as belonging to the same movement burst (i.e., a succession of steps). In order to accurately depict animal reactions to environmental cues and detect switching between behavioral states, we only considered bursts that were ≥50 fixes long (i.e., approx. corresponding to a four‐hour period), thus reducing the data to 172,068 steps. GPS fixes were processed into movement steps by connecting consecutive fixes within each burst, with the movement speed given as the length of line segments for each 5‐min segment (henceforth: *steps; S* in meters) and the directionality by the relative turning angle of the step in relation to the previous step (α in Radians).

Owing to the high temporal resolution of the data, the positional error of the fixes is assumedly large relative to the mean displacement within a step if the animal is moving slow or not moving at all (mean *S* of unprocessed data: 14.5 m). Accordingly, we strived to process the data to exclude steps with a high probability of representing “false movement,” that is, with large positional bias resulting from high positional inaccuracy while the animal remained relatively stationary (i.e., “encamped”). To this end, we employed a two‐step approach. We classified steps a) as belonging to an *active* or *resting* behavioral state based on accelerometer data and b) to represent *stationary* or *transient* movement behavior based on step length and turning angle. We then combined those classifications in order to identify “*resting*–*transient”* steps (a resting or passive animal for which the GPS data indicated large displacement), that likely represented steps in which positional error exceeded the actual distance moved by the animal (Figure 1c).

For a), that is, to separate active and resting behaviors, we used 3D acceleration data to classify fixes as either belonging to an *active* or a *resting* state using the developmental R package “activity tools” (Max Kröschel 2020 personal communication). Detailed information on activity classification is provided in Appendix S1. The approach discriminates active and resting states based on the physical activity that is displayed by an animal. Classes are distinguished by calculating dynamic thresholds based on smoothed activity data. Pointwise measurements of dynamic body acceleration data (i.e., the sum of acceleration on all axes) at three‐minute intervals were first smoothed by a moving window in order to account for behavior types with low acceleration during active phases (e.g., sitting still) or vice versa (e.g., scratching while resting). We selected a window width of 3 data points (i.e., 9 min) for smoothing and to identify active phases, based on visual examination of the data. The start and endpoints of the behavioral states (i.e., the timestamps) were then obtained from the resulting continuous measure by unsupervised classification based on the estimation of data‐ and species‐specific threshold values for active and resting behavior. We considered steps to belong to an *active* behavioral state, if both the start and end position of the step fell into a window of *active* behavior (defined by the start and end time stamp of the active phase) and as *resting* otherwise (Figure 1c). We included mixed steps with one active and one passive point within the passive category in order to err on the conservative side (i.e., as our main objective was to exclude “resting–transient” steps).

For b), that is, to separate stationary from transient movements, we employed hidden Markov models to classify steps as representing either a *stationary* or *transient* state of movement (Figure 1c). Steps were classified based on the distribution of step length (in meters) and relative turning angle (in Radians) in R package moveHMM (Michelot et al., 2016), assuming a gamma distribution for step length and a Von Mises distribution for the turning angles (i.e., considering both turning directions: left and right as ‐π ≤ α ≤ π). We then reconstructed the most probably sequence of movement states for the input data using the Viterbi algorithm as implemented in the package (Zucchini et al., 2017).

Finally, we classified all steps to belong to one of four types as the combination of *active* and *resting* behavioral states and *stationary* and *transient* movement state (e.g., *active*–*transient* for larger displacement within a bout of activity). We then excluded all steps that were classified as “*resting*–*transient”* from the data (18,698 steps), as they were considered to represent steps for which positional error exceeded displacement with high certainty. Ecologically, we considered the remaining three classes to correspond to the following behaviors: (1) long‐distance ground movement or flying, for instance between resources or sites (*active*–*transient*); (2) short‐distance movements such as during feeding, exploratory behavior, or predator avoidance behavior (*active*–*stationary*); (3) daytime or night‐time resting, for which most displacement may be attributed to GPS scatter (*resting*–*stationary*). This delivered 154,172 steps.

The periods over which data were available varied among individuals (between 50 and 400 days), owing to defective transmitters, movement out of the study area, or predation events. Accordingly, we finally cropped the dataset to cover the same time period in both years, including only the lekking and summer season (i.e., 2017 and 2018; hardly any data could be collected in the fall and winter of 2017/2018 due to hibernating solar tags). The final dataset comprised 153,370 steps from 13 animals covering the period between 23 April (first day) and 8 August (last day) in both years. For the period of active lekking (roughly mid‐April to mid‐May), we only had data from male capercaillie and thus pooled the sexes in the analysis. We expected no differences between the sexes with regards to movement in relation to environmental covariates.

### 2.4 Environmental predictors

We extracted all environmental covariates at the beginning of each step to depict the conditions that prompted a particular type of movement. All data were handled as raster files with a resolution of 25 × 25 m. We obtained information on land cover type and forest stand compositions from Swedish state forest inventory data (Lantmäteriet, 2017; Skogskarta, 2017; Skogsstyrelsen, 2018) as raster maps. With regard to forest stand composition, we considered the mean diameter at breast height (DBH) of all trees in a raster cell as well as the basal‐area‐weighted tree height in a raster cell (tree height). Land cover was classified into nine classes. We processed the land cover type “forest” into four classes based on tree composition in the raster cells, with forest being either dominated by Scots pine (≥75%) or Norway spruce (≥75%); forest with no dominant tree species and larger amounts of silver birch was classified as mixed forest. A small remainder of “forest” cells without inventory data on forest composition was classified as “unknown forest” (6.4% of all “forest” locations). Bogs in the study area were classified as either being located within the forest and featuring tree cover (“forest bogs”) or in open areas with little or no tree cover (“open bogs”). Clear‐cut areas in the forest matrix were classified dependent on their age, with clear‐cuts ≤5 years old and clear‐cuts >5 years old. Owing to the growth rate of trees we considered clear‐cuts ≤20 years old.

We quantified the influence of wind turbines in the wind farm on capercaillie movement by three different predictors: We (a) modeled turbine shadow flickering as the expected yearly amount (hours) of turbine shadow across the study area that was considered meteorologically plausible based on latitude, turbine specifications and average weather patterns in the software WindPRO 3.1 (EMD International A/S). For more information, see also (Coppes, Kämmerle, et al., 2020). In addition, we (b) predicted the number of visible wind turbines at each location in the study area based on terrain and vegetation heights (derived from high‐resolution aerial LiDAR data) which were validated using field observations (Nopp‐Mayr et al., 2021). Finally, we c) obtained Euclidean distances to the closest turbine access road (i.e., gravel forest roads that provide maintenance access to the turbine pads). We did not use the Euclidean distance to the closest turbine as it was highly correlated with turbine shadow (*r* > 0.6) and we considered turbine shadow and visibility to be the ecologically more meaningful predictors.

To depict daily and seasonal variation in movement behavior, we calculated the time of the day as a decimal number and the Julian day of the year relative to the 1st of January (range 113–220). Data were evenly spread around the longest day of the year 172 (i.e., 21st of June; sunrise to sunset 19 hr and 7 min; 56% of the data before 21st of June).

### 2.5 Analysis of movement speed and directionality

We analyzed movement speed *S* and relative turning angle α (i.e., pooling left and right turns as 0 ≤ α ≤ π) in generalized additive models in R package mgcv (Wood, 2011, 2017). This means that movement was fast for large values of *S* and directional for low values of α (i.e., small directional change relative to previous step). We assumed a gamma distribution for both response variables using a log‐link. Individual differences in mean step length were included as i.i.d. random effect. We modeled potential effects of within‐forest stand characteristics (i.e., mean DBH and tree height) and wind turbines (i.e., turbine shadow, visibility, and distance to turbine access roads) using thin plate splines with shrinkage, limiting the maximum flexibility of the splines to four degrees of freedom to prevent overfitting and ecologically nonmeaningful patterns. Daily and seasonal variation in movement behavior was included as a tensor product interaction of the covariates Julian day and daytime, with daily variation modeled with a cyclic cubic regression spline limited to five degrees of freedom and seasonal variation as a cubic regression spline with shrinkage limited to four degrees of freedom. We verified that model assumptions were met and assessed the final models for temporal autocorrelation in the residuals. There was no indication for residual autocorrelation of turning angles, but there was considerable autocorrelation in movement steps at lag one (i.e., the first consecutive step). Accordingly, we refitted the final model using the length of the previous step (*S*_t−1_) as a linear covariate, which effectively dealt with residual autocorrelation. For those steps without previous step (i.e., origins of bursts, steps after deletion of inaccurate steps; <3.4% of the data), we assigned the overall mean *S*. There remained no indication of residual autocorrelation in the model containing *S*_t−1_.

## 3 RESULTS

Mean movement speed *S* was 11.7 m per 5‐min interval (*SD* = 17.8m; range: 0.02–500 m). Mean turning angle α was 1.9 (*SD* = 0.96 Radian; range: 0–3.1 Radian).

Capercaillie movement speed was significantly related to land cover type, forest stand characteristics, wind turbine visibility, and the distance to turbine access roads, but not turbine shadow. The largest differences in movement directionality were related to land cover type, but there were minor effects of mean forest stand height and turbine shadow, but not turbine visibility and DBH. In addition, there was clear daily and seasonal variation in movement speed and movement directionality (Table 1).

**TABLE 1:** Model results of the GAMMs explaining capercaillie step length (a) and turning angle (b) for movement steps of 5‐min duration

(a) Model: step length *S* (speed)			
Predictor	Estimate	*SE*	*p*‐value
Intercept (LC – Clear‐cut <5 year old; reference)	1.891	0.029	<.001
LC – Clear‐cut >5 year old	0.120	0.023	<.001
LC – Cultivated land	0.604	0.300	.041
LC – Forest bog	0.195	0.016	<.001
LC – Open bog	0.380	0.035	<.001
LC – Mixed forest	0.240	0.014	<.001
LC – Unknown forest	0.230	0.020	<.001
LC – Pine forest	0.240	0.014	<.001
LC – Spruce forest	0.222	0.022	<.001
Length previous step *S*_t‐−1_	0.026	<0.001	<.001
**Predictor**	**Edf**		***p*‐value**
Number of visible turbines	2.29		<.001
Turbine shadow	0.80		.211
Distance access roads	1.42		.010
Daytime * Julian date	8.79		<.001
Stand mean DBH	1.10		<.001
Stand mean height	2.00		<.001
**(b) Model: turning angle α (directionality)**			
**Predictor**	**Estimate**	***SE***	***p*‐value**
Intercept (LC – Clear‐cut <5 year old; reference)	0.628	0.008	<.001
LC – Clear‐cut >5 year old	0.013	0.008	.113
LC – Cultivated land	−0.720	0.116	<.001
LC – Forest bog	0.019	0.006	<.001
LC – Open bog	−0.096	0.013	<.001
LC – Mixed forest	0.034	0.005	<.001
LC – Unknown forest	0.037	0.008	<.001
LC – Pine forest	0.025	0.005	<.001
LC – Spruce forest	0.032	0.009	<.001
**Predictor**	**Edf**		***p*‐value**
Number of visible turbines	≈ 0		.670
Turbine shadow	1.92		.016
Distance access roads	1.60		.011
Daytime * Julian date	7.86		<.001
Stand mean DBH	≈ 0		.400
Stand mean height	0.36		.060

Note

Coefficient estimates, their standard errors (*SE*), and *p*‐values are provided for categorical predictors (Land cover LC) and linear predictors; effective degrees of freedom and *p*‐values for smooth terms.

### 3.1 Habitat characteristics

**FIGURE 2:**
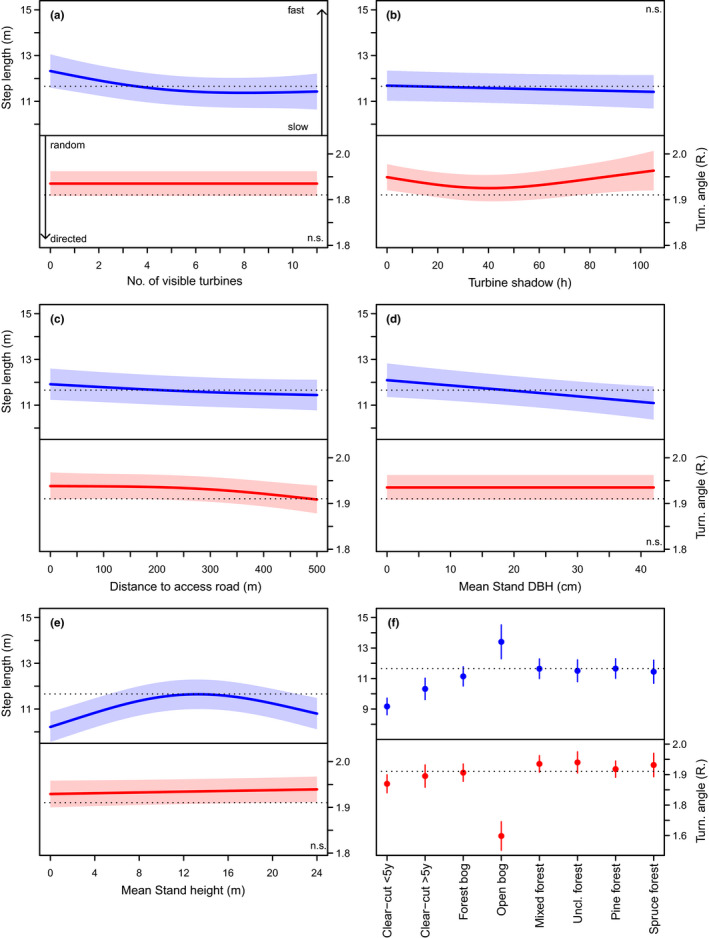
Effect plots displaying covariate effects of the GAMMs explaining variation in step length (blue) in meters per 5‐min interval and turning angle (red) in Radian as a function of wind turbine predictors, forest stand characteristics, and land cover. Larger values for step length denote higher movement speed and small values a higher degree of stationarity, while small values for turning angle denote higher directionality of movement and large values a higher degree of undirected movement or GPS scatter during stationarity (see A). All other covariates were held at their mean. The dotted black line is the overall mean. Note that panel F excludes the category “cultivated land,” for which sample size was small and SEs large (see Table 1). Nonsignificant effects are marked as “n.s.” in the plots (see Table 1)

Capercaillie mean movement speed was fastest and most directional on open bogs. It was slowest, but still directional on clear‐cuts, with recently cut areas being associated with slower movement speed and higher directionality than areas cut >5 years ago (Figure 2). There was no difference in movement speed between forest stand types (e.g., spruce, pine, mixed stands), for which mean movement speed fell on the overall average. Movement in forest was less directional as compared to open habitat types, but slightly more directional on forest bogs (Figure 2). Movement speed decreased with increasing DBH and was thus higher in younger, denser stands than in more mature stands. Movement speed peaked at intermediate stand heights, whereas directionality decreased slightly with increasing stand height (Figure 2).

In addition, capercaillie movement speed mainly decreased with increasing turbine visibility up to ≥6 turbines, but also with increasing distance to turbine access roads, where movement was also more directed (approximately ≥200 m; Figure 2). Although movement speed was unaffected by turbine shadow, movement was more directional at intermediate values.

### 3.2 Daily and seasonal variation

**FIGURE 3:**
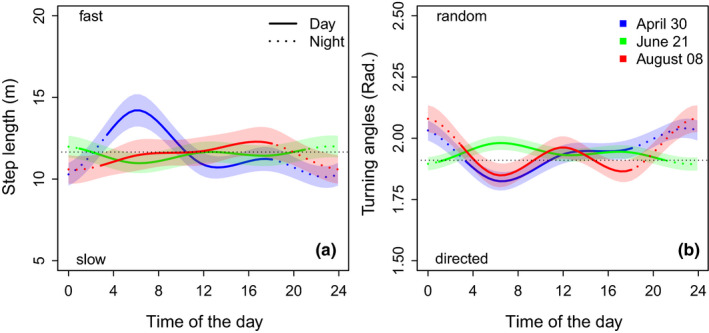
Effect plots displaying variation in step length (left) and turning angle (right) as a function of the time of the day at three times during the year. The occasions represent the peak in capercaillie lekking activity (blue; note that only male animals provided data during this period), the summer solstice (green, i.e., the longest day), and late summer (i.e., the end of the study period). Larger values denote higher movement speed (left) and low directionality (right), while low values accordingly depict more stationarity (left) and high directionality in movement (right). Dotted lines indicate times before sunrise and after sunset. All other covariates were held at their mean

There was clear diurnal variation in movement speed, and this variation was influenced by seasonality. Diurnal variation in movement directionality matched variation in movement speed (Figure 3). During the lekking season, capercaillie males displayed a morning peak of increased movement speed and directionality (i.e., a minimum in α) between approximately 4 and 8 a.m. UTC time (6 and 10 a.m. local time). This was followed by more random movement with speeds on the overall mean during the reminder of the day and a marked reduction in movement speed and directionality during the night (Figure 3). This daily pattern disappeared toward the day of the summer solstice (i.e., longest day), on which there was no clear pattern in daily mean movement speed. As days shortened toward late summer, nocturnal minima in movement speed and directionality increased (i.e., a maximum in α), with overall higher speeds during morning and a peak in movement speed and directionality in the afternoon (Figure 3).

## 4 DISCUSSION

In our study, we applied a two‐step approach to analyze movement behavior of a ground‐dwelling bird species, the capercaillie, in a Swedish wind farm in response to environmental and seasonal cues using high‐resolution GPS tracking data (i.e., 5‐min sampling regime).

### 4.1 Daily and seasonal variation in movement

The strongest effect on movement speed was related to capercaillie lekking activity (Figure 3). At this time of the year (i.e., late April and early May), movement speed peaked during morning hours just after sunrise, followed by a constant movement speed on the overall average during the afternoon. Morning movements were highly directional, whereas movement was neither directed nor random in the afternoon (Figure 3). Previous work on movement behavior of lekking capercaillie males revealed a daily periodicity in movement around lekking sites, where males left the lek in the late morning to quickly move to daytime ranges, from where they slowly diffused back to the lek or its vicinity throughout the afternoon (Wegge & Larsen, 1987; Wegge et al., 2013). In addition, capercaillie form “exploded leks,” in which males perform spontaneous fast movements of up to several hundred meters into a competitor's territory in the early morning hours (Wegge et al., 2013). We thus suggest that the pronounced morning peak in directed fast movement during this time (Figure 3) corresponds to such quick intralek movements and movements to the daytime ranges in the late morning after lekking activity may have ceased, whereas the average movement speed and turning angle during the afternoon indicates switching between foraging, rest, and slow movement as could be expected for animals slowly diffusing back to the lek's vicinity before nightfall.

In both spring and late summer (April, August), movement speed was minimal and highly undirected during darkness hours (i.e., likely indicating GPS scatter during a bout of inactivity; Figure 3). Capercaillie roost on trees during the night to minimize predation (Klaus et al., 1989) and this behavior is clearly reflected by our results. However, this was not detectable around midsummer (the longest day of the year), when no clear daily patterns in movement speed were detectable, although movement was least directional in the mornings. This lack of a pattern indicates a continuous, unstructured switching between active and passive behaviors throughout the day, without a clearly pronounced night‐time rest (i.e., as there is hardly any darkness). This is supported by direct analysis of the tags’ accelerometer data, which indicates an unstructured succession of active and passive phases during midsummer, while in spring and late summer “nights” are predominately characterized by passive phases (unpublished data of the authors). Bird species at high latitudes, both diurnal and nocturnal, may adapt their behavioral strategies to the extreme photoperiod, in order to fully capitalize on the available day‐ or night‐time hours (Daan, 1981; Daan & Aschoff, 1975; Sanz et al., 2000; Zárybnicka et al., 2012). To our knowledge, we are the first to describe seasonal shifts in activity patterns in relation to the photoperiod for capercaillie and its pronunciation is likely to also vary with latitude.

As days shortened, the birds returned to a clearly pronounced day and night rhythm. In contrast to the lekking season, in late summer there was an afternoon peak in fast‐directed movement, with an identifiable bout of slower, but also very directed movement in the morning. This may indicate that birds left their night‐time roosting sites in the morning primarily to feed and slowly move between resources (i.e., slow but continuous movement), while movement was faster in the later afternoon, likely indicating between‐site movement or a return to night‐time roosting sites. The early afternoon was characterized by undirected movement at average speeds, potentially indicating switching between feeding and resting bouts (i.e., with undirected GPS scatter).

### 4.2 Movement through different habitat types

With regard to environmental covariates, we found the largest effects for the type of land cover. Capercaillie movement on open bogs (and cultivated land) was considerably faster and more directed than in all other habitat types, implying that capercaillie quickly cross and leave these habitats. Open bogs represent, apart from recent clear‐cuts, the most open habitat types present in the forest matrix. Capercaillie may minimize predation risk by avoiding or quickly crossing such habitat types, because predation risk is elevated in open areas with little cover (Kvasnes & Storaas, 2007). Perhaps surprisingly, this was not reflected by fast‐directed movements on clear‐cuts. Recent clear‐cuts (≤5 years old) were rather associated with the slowest, yet still directed movement patterns, suggesting a slow, but rather purposeful movement. This may indicate that clear‐cuts and open bogs are used to different ends. While open bogs may simply be crossed rapidly, clear‐cuts are typically smaller units within the forest matrix that may offer attractive forage and edge habitats. Capercaillie utilize clear‐cuts in summer (Storch, 1993) and also select inner forest edges (Hofstetter et al., 2015), which are present on the edge of clear‐cuts. Finally, slower movement speed on clear‐cuts may also be related to movement resistance if recent clear‐cuts are associated with large amounts of coarse woody debris. Although there was no difference in movement speed and directionality between categorical forest stand types (e.g., pine or spruce dominated stands), there was variation in movement speed but not directionality within stands that was related to stand density and height. Capercaillie moved fastest in stands with lowest mean DBH and stands of intermediary height (e.g., young, but already closed stands), while they remained longest (i.e., moved slowest) in mature stands (large height and DBH), but also in very young stands (minimal height), which likely reflects their slow movement in and around recent clear‐cut areas (compare Figure 2e,f).

In addition, we detected a decrease in movement speed in relation to wind turbine visibility, implying that birds made smaller steps and most likely spend more time in one position (or in a small area) as more turbines were visible. A reduction in movement speed together with being inconspicuous is a known predator avoidance strategy in capercaillie (Klaus et al., 1989). We suggest that the visual cues provided by an increasing number of visible turbines add up to provoke an increasing anti‐predator response in the birds (Figure 2). The interpretation of our findings regarding turbine shadow is, however, less straightforward. A higher incidence of low turning angles (i.e., moving straighter) in combination with a step length on the overall mean may indicate that capercaillie display normal movement in areas that receive little shadow. By contrast, high turning angles (i.e., turning around) together with slightly lower movement speeds are more indicative of GPS scatter, which suggests that capercaillie spend more time being stationary, which might support a predator avoidance response. A behavioral avoidance of areas that receive a high degree of wind turbine effects (e.g., shadow, noise, visibility) has already been demonstrated for capercaillie in Sweden (Taubmann et al., 2021) and elsewhere, with no indication for habituation (Coppes, Kämmerle, et al., 2020). We thus supplement previous findings on turbine effects with a more mechanistic understanding, indicating that the use of strongly affected areas may be related to higher costs of coping with this stressor.

### 4.3 Limitations

Through the application of a two‐step analysis, we successfully inferred a number of behavioral reactions and movement decisions from noisy data. We employed a simple method to classify activity states from auxiliary data (i.e., tag acceleration) and combined this information with characteristics of the movement steps to identify steps with a high probability of large positional error, thus increasing the precision of the data. The majority of GPS tags nowadays have the capability to collect such auxiliary data. Nonetheless, despite clear seasonal variation in daily movement patterns, the ecological interpretation of speed and directionality in relation to environmental covariates still remains somewhat vague. Using movement data, we are only capable to quantify the reaction of an animal in terms of speed and angle. While it appears plausible that fast and directed movements depict moving between resources or flight, and slow and random movements depict stationary behaviors such as feeding or resting, the identification of the actual behavior remains obscure. In order to do so, and make interpretations truly valid, field observations of birds carrying the transmitters would be needed (as is e.g., done for the classification of acceleration data; Kröschel et al., 2017). This is, however, unrealistic for cryptic, forest‐dwelling species, but might be achieved using captive individuals. Nonetheless, combining the analysis of movement speed and angle with information on the actual behavioral state for instance through classification of acceleration data may present a promising approach for future applications.

Another uncertainty concerns the question of how to scale up the population‐level consequences of movement (Hebblewhite & Haydon, 2010; Morales et al., 2010), for instance, with regard to our findings on turbine visibility (Figure 2). Wind turbines negatively affect habitat selection in capercaillie (Coppes, Kämmerle, et al., 2020; Taubmann et al., 2021). However, this is arguably insufficient as proof that slower movement corresponds to a negative effect, for example, owing to increased predator avoidance behavior. A convincing argument might as well be made that the birds' reaction is seemingly adequate to cope with this stressor, given the presence of active lekking sites in and around the windfarm. Accordingly, the long‐term consequences for the birds' fitness, and hence the population, remain unclear.

## 5 CONCLUSIONS

Through the analysis of high‐resolution tracking data, we obtained interesting and novel insights into the seasonal and environmental correlates of capercaillie movement decisions. We also supplement previous behavioral observations on lekking behavior and wind turbine avoidance with a more mechanistic view. However, the use and interpretation of movement speed and directionality heavily relies both on a sound basic knowledge of the target species' ecology and the ecological context, as well as the collection of fine‐scale data without sacrificing tag lifetime (e.g., using rechargeable solar‐tag). If either of these prerequisites cannot be met, researchers should critically evaluate the information gain that they expect from a high‐resolution sampling regime given their study objectives.

## CONFLICT OF INTEREST

None declared.

## AUTHOR CONTRIBUTIONS

**Jim‐Lino Kämmerle:** Conceptualization (equal); data curation (equal); formal analysis (equal); visualization (lead); writing–original draft (lead); writing–review and editing (equal). **Julia Taubmann:** Funding acquisition (equal); investigation (lead); project administration (equal); resources (equal); writing–review and editing (equal). **Henrik Andrén:** Funding acquisition (equal); investigation (equal); project administration (equal); resources (equal); writing–review and editing (equal). **Wolfgang Fiedler:** Investigation (equal); methodology (equal); writing–review and editing (equal). **Joy Coppes:** Conceptualization (equal); funding acquisition (equal); investigation (equal); project administration (lead); resources (equal); supervision (lead); writing–review and editing (equal).

## Supporting information

Supplementary Material

## Data Availability

The data associated with this study are available in the Dryad data repository: https://doi.org/10.5061/dryad.wpzgmsbnc.
