# Supplementary material for: Environmental and seasonal correlates of capercaillie movement traits in a Swedish wind farm
Source: Ecol Evol. 2021 Aug 5;11(17):11762–73. doi: 10.1002/ece3.7922 (PMC8427587; doi:10.1002/ece3.7922)
Supplement: Supplementary file 1 — Supplementary Material [file ECE3-11-11762-s001.docx]

**Supplementary Material**

**Environmental and seasonal correlates of capercaillie movement traits in a Swedish wind farm**

**Authors**

Kämmerle, J.-L.^1,2^, Taubmann, J.^1,2^, Andrén, H.^3^, Fiedler, W.^4^, Coppes, J.^1*^

1: FVA Wildlife Institute, Forest Research Institute of Baden-Wuerttemberg FVA, Wonnhaldestr. 4, 79100 Freiburg, Germany

2: Chair of Wildlife Ecology and Management, University of Freiburg, Tennenbacher Str. 4, 79106 Freiburg, Germany

3: Grimsö Wildlife Research Station, Department of Ecology, Swedish University of Agricultural Sciences, SE-739 93 Riddarhyttan, Sweden

4: Department of Migration and Immuno-Ecology, Max Planck Institute of Animal Behavior, Am Obstberg 1, 78315 Radolfzell, Germany

*Corresponding author: joy.coppes@forst.bwl.de

**ORCID ID:**

Jim-Lino Kämmerle: <https://orcid.org/0000-0002-5751-9757>

Julia Taubmann: <https://orcid.org/0000-0002-8339-0184>

Henrik Andrén: <https://orcid.org/0000-0002-5616-2426>

Wolfgang Fiedler: <https://orcid.org/0000-0003-1082-4161>

Joy Coppes: <https://orcid.org/0000-0002-5295-8638>

**Supplementary Material S1: Additional information on activity classification**

We analysed the 3D-acceleration data collected by the tags to classify GPS fixes as either as *active* or *resting* class using the developmental R package ‘activity tools’ (Max Kröschel 2020 personal communication). This was the first step in the pre-analysis of the data designed to exclude steps with a high probability of representing ‘false movement’, i.e. with large positional bias resulting from high positional inaccuracy while the animal remained relatively stationary (i.e. ‘encamped’).

Here we provide more detailed information on the method to classify activity data into two classes and subsequently assign this information to GPS location data. The approach discriminates active and resting classes based on the sum of the physical activity that is displayed by an animal based on the acceleration data. This approach is grounded on the assumption the active class is characterized mainly by active behaviours (i.e. higher body acceleration) and vice versa.

**Distinguishing activity states classed based on acceleration data**

Activity classes (*active* and *resting*) are distinguished by calculating dynamic thresholds based on smoothed acceleration data (i.e. the sum of the physical activity). Henceforth, active behaviours are those that involve repeated motions of the animal (for example feeding and walking), while resting behaviours include all those during which the animal is predominantly motionless (for example lying and standing). We collected acceleration data at three minute intervals for 10 seconds each. We analysed dynamic body acceleration data (i.e. the sum of acceleration on all three axes). To account for the fact that animals also display inactive behaviours that have low activity values during an active class (e.g. a short bout of vigilance behaviour while walking), these pointwise measurements were smoothed by a moving window in order to account for behaviours with low acceleration during active phases (e.g. sitting still) or vice versa (e.g. scratching while resting). After visual examination of the data, we selected a window width of 3 data points (i.e. 9 minutes) for smoothing. The start- and endpoints of the behavioural states (i.e. the timestamps) were then obtained from the resulting continuous measure by unsupervised classification based on the estimation of an optimal data- and species-specific threshold value that separates active and resting behaviour. The timestamps at which the smoothed activity crosses the estimated threshold were exported as the start and end points of an active state, respectively (see Figure S1). Hence, a phase of active or resting behaviour is defined by the time interval between the two consecutive time stamps at which the threshold is crossed and is always ≥ 9 minutes in length (i.e. 3 data points).

**Assigning activity classes to movement steps (GPS fixes)**

We considered movement steps to belong to an *active* behavioural state, if both GPS positions of the step (i.e. the start- and end positions) fell into a time window classified as *active* and as *resting* otherwise (Fig. 1C). Our main objective was to identify those movement steps that did not represent real movements (but GPS error), because the animal was in a resting state and thus not moving. We therefore included mixed steps with one active and one passive point within the resting category in order to err on the conservative side (i.e. rather than including a larger number of potentially “false movements”).


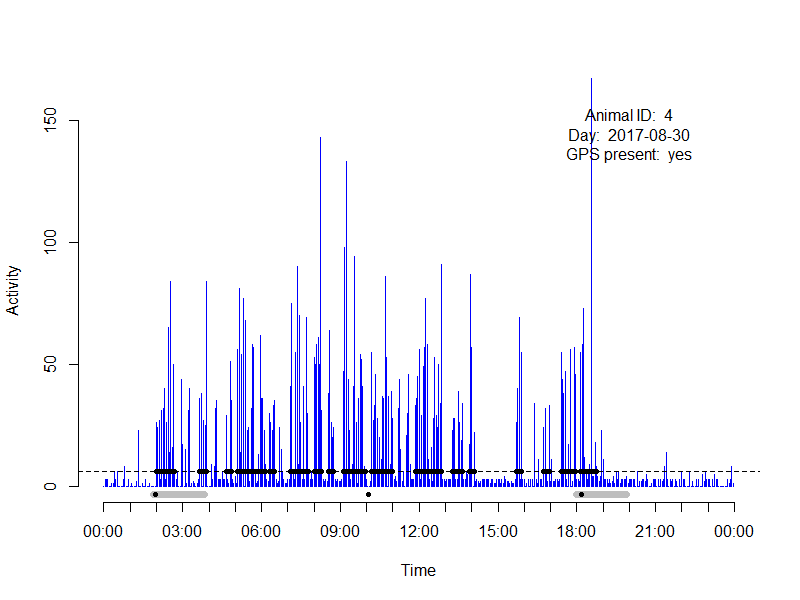


**Supplementary Figure S1:** Exemplary depiction of 24 hours of dynamic body acceleration data (i.e. the sum of all three axes; blue bars) and the classification into *active* (black fat line) and *resting* behaviour types (black dashed line). Raw activity data at individual pointwise measurements were smoothed by a moving window of 9 minutes in width (i.e. 3 data points) and subsequently classified into active and resting phases using unsupervised classification. GPS points (black dots above x-axis) were then assigned to the respective activity state according to their time stamp. Note that we included only 3 GPS points for better readability of the figure, while the actual sampling interval for GPS fixes was 5-minutes. Grey shading denotes crepuscule hours in UTC time.
